# Translation and Cross-Cultural Adaptation of the Supportive and Palliative Care Indicators Tool into Japanese: A Preliminary Report

**DOI:** 10.1089/pmr.2021.0083

**Published:** 2022-08-18

**Authors:** Ai Oishi, Jun Hamano, Kirsty Boyd, Scott Murray

**Affiliations:** ^1^ Primary Care Research Unit, Graduate School of Health Data Science, Yokohama City University, Yokohama, Japan.; ^2^ Division of Clinical Medicine, Faculty of Medicine, University of Tsukuba, Tsukuba, Japan.; ^3^ Primary Palliative Care Research Group, User Institute, University of Edinburgh, Edinburgh, United Kingdom.

**Keywords:** identification, palliative care, primary care, SPICT, translation

## Abstract

**Background:**

There is a need for tools in primary care to support clinicians to identify patients with unmet palliative care needs. The Supportive and Palliative Care Indicators Tool (SPICT) is concise and covers most conditions in primary care settings. However, the SPICT was not available in Japanese.

**Methods:**

The translation and cultural adaptation of the SPICT was conducted in four stages: forward translation (Stage I), synthesis (Stage II), back translation (Stage III), and expert committee review (Stage IV).

**Results:**

During the translation process, any content challenging to translate was addressed in Stage II and through discussion among the researchers. The expert committee review provided valuable insights on palliative care in Japan in addition to the translation.

**Conclusion:**

The Japanese version of the SPICT and its user guide are ready to be tested in clinical settings. They have the potential to help Japanese family physicians integrate palliative care in their care of patients with all life-limiting illnesses.

## Introduction

Primary care teams should be able to identify patients who might benefit from receiving a palliative care approach.^1^ However, it is unclear which patients, when in the illness, and to what extent palliative care should be considered.^2,3^ This is also the case in Japan where the number of deaths is rising^4^ while frailty and nonmalignant chronic diseases are increasingly prevalent.^5^

Several tools have been developed for primary care clinicians to promote systematic identification of people with unmet palliative care needs.^1,6,7^ Among them, the Supportive and Palliative Care Indicators Tool (SPICT)^8^ is a one-page tool suited to busy clinical settings and covers most conditions that clinicians encounter in primary care settings. The SPICT has been translated into several languages,^9–12^ but was not available in Japanese. The SPICT aims to let clinicians identify all patients who might benefit from primary or specialist palliative care. Where the clinicians have adequate training in palliative care, most patients identified can be treated by the clinicians which could be generalists such as family physicians. So, the SPICT helps generalists identify patients they can provide a palliative care approach to. When they do not have the training, or they need specialist advice then they can refer.

Our objective was to translate and culturally adapt the SPICT so that we could use it in Japanese primary care. This article describes the preliminary results of this project: the translation and expert committee review of the Japanese version of the SPICT (SPICT-JP). This project was done as part of a larger project, which aims to understand family physicians' views on identifying patients for palliative care.

## Methods

### Translation and cross-cultural adaptation of the SPICT

**FIG. 1.**
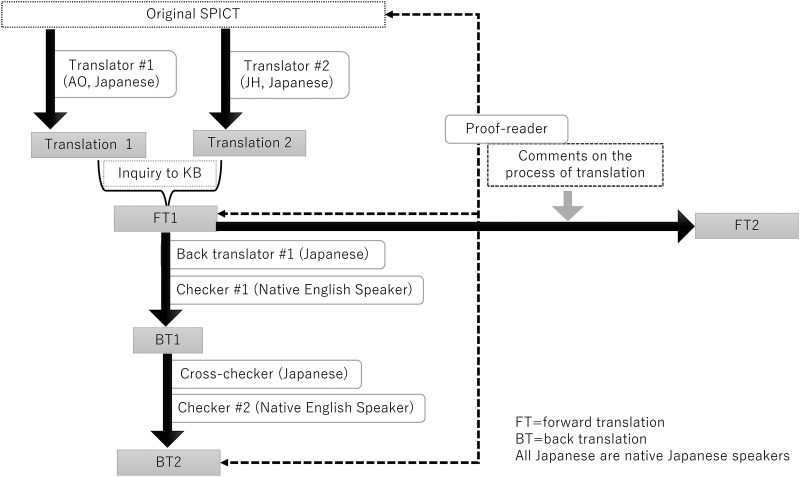
Process of translation.

We referred to international guidelines and literature reviews for translation and cross-cultural adaptation of health-related quality of life measures,^13–16^ and took the following four stages (Fig. 1 for the first three stages).

### Stage I: translation

Two researchers (A.O. and J.H.) translated the original English version of the SPICT into Japanese independently. Both had clinical experience in community palliative care and knew how words and phrases would be understood by family physicians in Japan. In translating the SPICT into Japanese, problematic and unclear expressions were recorded for consideration in Stage II.

### Stage II: synthesis

The two translations were synthesized. We consulted the original developer (K.B.) about unclear expressions to seek a fluent and accurate translation. For instance, during this process, a question was raised regarding the sentence: “Patients ask for supportive and palliative care, or treatment withdrawal.” One translator thought that most patients do not make such requests. He thought that in Japan, family members do so on their behalf and a family request rather than a patient request could be a sign of a deterioration in health. The developer and translators agreed that this was an example of important cultural differences. The expert committee members provided additional expertise in such aspects of translation in Stage IV.

### Stage III: back translation

The synthesized version of the translation (FT1) was back translated by a Japanese professional translator ( = back translator) and checked subsequently by a native English speaker ( = checker no. 1). This back translation (BT1) was then checked by another Japanese professional translator ( = cross-checker) referring to the original version for semantic equivalence, followed by a further review by a second native English speaker ( = checker no. 2). This process produced the final back translation (BT2). An independent professional proof reader with English competency compared the original SPICT, FT1, and BT2, and provided a report. We reviewed this report to develop FT1 into FT2.

### Stage IV: expert committee review

**FIG. 2.**
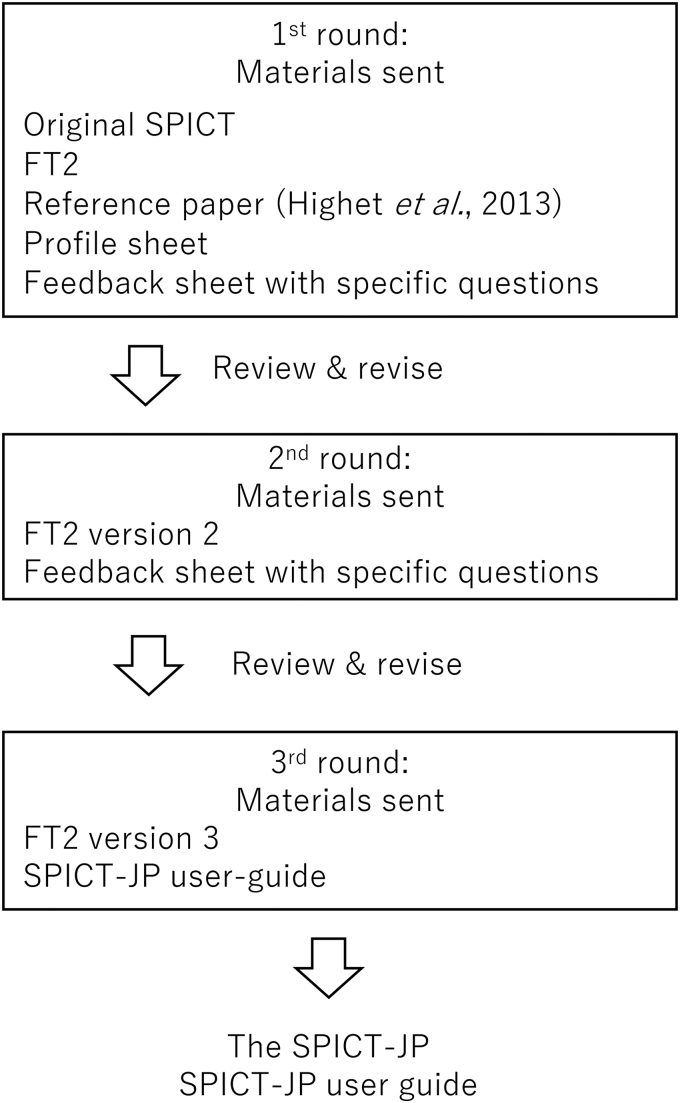
Overview of the expert committee review.

The expert committee consisted of eight doctors, including family physicians, palliative care specialists, and homecare specialists who do home visits for frail and unwell patients at the patient's own home (Supplementary Data S1). All members were asked to answer specific questions (Supplementary Data S2) and provide feedback about FT2. An amended FT2 and some specific questions (Supplementary Data S3) were sent back to the expert committee members for further review. The feedback on the amended FT2 and answers to those specific questions were used to create a final FT2. Figure 2 describes the overview of the process.

### Developing a SPICT-JP user guide

One of the recommendations from the expert committee review was to provide more information about the SPICT-JP for users. Thus, we decided to develop a Japanese user guide, which would be simple (one A4 sheet) and reflect the brevity of the SPICT itself. The user guide was drafted by referring to the comments from the expert committee, user guides of the SPICT in English and a Japanese article on how to open end-of-life conversations.^17^ The first draft was circulated to the expert committee for their feedback. The second version was reviewed again, and no modifications were recommended.

## Results

The final version of the SPICT-JP and its Japanese user guide are available in Supplementary Data S5. The user guide contains the following:

1. Purpose of the SPICT-JP
2. What the SPICT-JP is not designed for (e.g., prognostication or automatic referral to specialist palliative care)
3. Situations where the SPICT-JP can be used (e.g., screening patients, multidisciplinary meetings, and joint meetings with specialists)
4. Actions to take after the identification of patients with examples of how to open end-of-life conversations.

### Obtaining equivalence

The most challenging points to translate are shown in Table 1. These were resolved by discussion among researchers and through seeking opinions from the expert committee. An example of this was an item on ventilation in respiratory diseases. The full meaning of “ventilation is contraindicated” in the original SPICT assumed there had been an assessment of poor outcomes in relation to prognosis and/or quality of life. This was not understood clearly by some of the expert committee members.

**Table 1.** Words and Phrases Discussed in the Translation Process

Stages	Expressions (italicized) needed to be discussed	Relevant equivalence (Guillemin et al^20^)
II	No longer able to communicate using *verbal language*	Semantic (vocabulary)
II	Depend on others for most *care needs* due to.	Semantic (vocabulary)
II	Patient *asks for* supportive and palliative care, or.	Idiomatic, experiential, conceptual
II	Persistent, *troublesome* symptoms:	Semantic (vocabulary), conceptual
IV	*Supportive* and palliative care needs	Semantic (vocabulary), conceptual
IV	: and;	Semantic (grammatical)
IV	*Ventilation is contraindicated*	Conceptual
IV	Agree current and future care goals, and a *care plan* with the person and their family	Conceptual
II and IV	*Too frail for oncology treatment*.	Semantic, idiomatic, conceptual
II and IV	Too *frail* for oncology treatment.	Semantic (vocabulary)
II and IV	*Optimal treatment* of underlying condition(s)	Conceptual

It might be related to the differences in clinical practice between other countries and Japan where long-term ventilation is much more common. Japanese doctors found it difficult to imagine situations in which ventilation was contraindicated without any background information being specified. We replaced the sentence with “ventilation is contraindicated because it would not improve patient prognosis or quality of life” and asked the expert committee members for their views on this in the second round (Supplementary Data S3). Their responses to this replacement expression were favorable.

In addition, some direct translations of English terms were thought not to fit with Japan and were paraphrased to align with Japanese health care. One example was the phrase “care planning.” In the Japanese long-term care insurance scheme, the term “care plan” referred specifically to the long-term care service plan. So, we replaced the word with “plan.” Another example was “supportive and palliative care.” Some members said that “supportive care” was not a recognized term in Japan and one doctor suggested omitting the word “supportive.” After careful consideration, we decided to keep the word “supportive.” We felt it would be understood particularly for those working in palliative care, and it would be better to keep the original SPICT phrases whenever possible.

### Feedback about the SPICT-JP

Although the purpose of the expert committee was to secure an accurate translation of the original SPICT, the members also provided valuable insights on the language and content of SPICT-JP and how it should be operationalized in Japan. Some concerned about unclear criteria within the SPICT. Also, there were some confusions about who should use the SPICT-JP in Japanese clinical settings. Others raised general issues on palliative care in Japan in relation to the identification of patients who may be needing palliative care. These points might be related to that there are no standard criteria for referral to specialist palliative care or standard definition of generalist/specialist palliative care. The details of their feedback are in Supplementary Data S4.

## Discussion

During the process of forward and back translation, some issues for discussion were identified. However, Stage IV, when we obtained views from the expert committee, played a key role in the adaptation process. Comments from the committee were informative and valuable in augmenting Stages I to III. A review of guidelines for cross-cultural adaptation of questionnaires maintained that inclusion of an expert committee is crucial, while questioning the value of a back translation.^18^ In addition, a study comparing back translation with an expert committee showed that the committee contributed substantially toward a better translation and cross-cultural adaptation.^19^ The authors suggested that back translation may have a limited role, which is in line with our experience.

To maximize the role of an expert committee review, multidisciplinary input has been recommended.^13,20,21^ However, we recruited expert committee members to represent doctors who were working in relevant clinical practice similar to the target group for implementation of SPICT-JP. Having other professionals and doctors from other specialties could have produced wider and different insights and perspectives. Further consultation with more diverse professionals, as was done in developing Thai version of SPICT,^9^ will be considered to improve the SPICT-JP in the future.

The expert committee provided valuable advice concerning general issues relating to palliative care in Japan. Unfortunately, it was impossible to integrate all of this in the final version of the SPICT-JP due to the specific role of the expert committee review, but they did inform the user guides.

Several questions about how the SPICT-JP should be used in Japan were raised by the expert committee members as shown in Supplementary Data S4. However, it could be claimed that it was not the tool but the users (Japanese clinicians) who should define how the tool would be used. Further investigation is needed to decide how the SPICT-JP should be implemented in Japan.

In conclusion, we completed the translation and cross-cultural adaptation of the SPICT. The Japanese version of the SPICT and its user guide (Supplementary Data S5) are now ready to be tested in clinical settings. They have the potential to help Japanese family physicians integrate palliative care in their care of patients with all life-limiting illnesses.

## Supplementary Material

Supplemental data

Supplemental data

Supplemental data

Supplemental data

Supplemental data

## Abbreviation Used

BT1, BT2: back translation
FT1, FT2: synthesized version of the translation
SPICT: Supportive and Palliative Care Indicators Tool
SPICT-JP: Japanese version of the SPICT
