# Supplementary material for: Translation and Cross-Cultural Adaptation of the Supportive and Palliative Care Indicators Tool into Japanese: A Preliminary Report
Source: Palliat Med Rep. 2022 Aug 18;3(1):1–5. doi: 10.1089/pmr.2021.0083 (PMC9438437; doi:10.1089/pmr.2021.0083)
Supplement: Supplemental data [file Supp_DataS1.docx]

**Supplements**

**Supplement 1. Profile of expert committee members**

| No | Affiliation | Background |
| --- | --- | --- |
| 1 | University | Family physician with special interests in medical education and palliative care |
| 2 | Metropolitan cancer centre | Palliative care specialist, originally trained in internal medicine |
| 3 | Community hospital (160 beds) | Palliative care doctor, originally trained in family medicine |
| 4 | Home care department at a hospital (>800 beds) | Home care doctor, originally trained in family medicine |
| 5 | Palliative care department at a hospital (>800 beds) | Palliative care specialist and home care doctor, originally trained in internal medicine |
| 6 | Home care clinic | Home care specialist, originally trained in internal medicine |
| 7 | Family medicine clinic | Family physician |
| 8 | Family medicine clinic | Family physician with special interests in infectious diseases |
