# Supplementary material for: Translation and Cross-Cultural Adaptation of the Supportive and Palliative Care Indicators Tool into Japanese: A Preliminary Report
Source: Palliat Med Rep. 2022 Aug 18;3(1):1–5. doi: 10.1089/pmr.2021.0083 (PMC9438437; doi:10.1089/pmr.2021.0083)
Supplement: Supplemental data [file Supp_DataS2.docx]

**Supplement 2. Specific questions sent at the 1^st^ round of the expert committee review**

| \| 1. Capacity: would it be acceptable to translate capacity into ‘意思決定能力 *ishi-kettei-nouryoku* (a capacity for making a decision)’? 2. Coordinate: Given the word ‘コーディネート *ko-dine-to*’ is now widely accepted in Japan, it might be better to translate the term into ‘コーディネート *ko-dine-to*’ rather than ‘調整 *cho-sei*’(the direct translation). What do you think? \| \| --- \| |
| --- | --- |
