# Supplementary material for: Translation and Cross-Cultural Adaptation of the Supportive and Palliative Care Indicators Tool into Japanese: A Preliminary Report
Source: Palliat Med Rep. 2022 Aug 18;3(1):1–5. doi: 10.1089/pmr.2021.0083 (PMC9438437; doi:10.1089/pmr.2021.0083)
Supplement: Supplemental data [file Supp_DataS3.docx]

**Supplement 3. Specific questions sent at the 2^nd^ round of the expert committee review**

| \| 1. Do you think it is better to have a specific cut-off for a ‘low BMI’? 2. A comment was raised questioning the translation of ‘functional ability’. We were unable to find any other translation. Do you have any alternatives for this? Or do you think the current translation is OK? 3. An opinion was raised that allied health professionals might not understand the word ‘frail’. The usage of this word is based on the statement from the Japan Geriatrics Society. Do you have any comments on this? 4. In respiratory diseases, do you think it is better to say that ‘they are not suitable for ventilation anymore’? The original sentence, ‘ventilation is contraindicated’ means ‘ventilation is contraindicated because it does not improve either prognosis or quality of life’. 5. Care planning: an opinion was raised that the term ‘care planning’ could be confused with the term ‘care plan’ used in the long-term care insurance scheme. Therefore, it was changed to ‘ケア計画 kea-keikaku’. What do you think? 6. Regarding performance status, weight loss, the degree of independence of daily living and the first item in the neurological disease (‘Progressive deterioration in physical and/or cognitive function despite optimal therapy’), some said there were patients who were relatively stable even though they met these criteria. What do you think? \| \| --- \| |
| --- | --- |
