# Supplementary figures and images for: Translation and Cross-Cultural Adaptation of the Supportive and Palliative Care Indicators Tool into Japanese: A Preliminary Report
Source: Palliat Med Rep. 2022 Aug 18;3(1):1–5. doi: 10.1089/pmr.2021.0083 (PMC9438437; doi:10.1089/pmr.2021.0083)

**Supplement 5. The SPICT-JP and its user-guide**


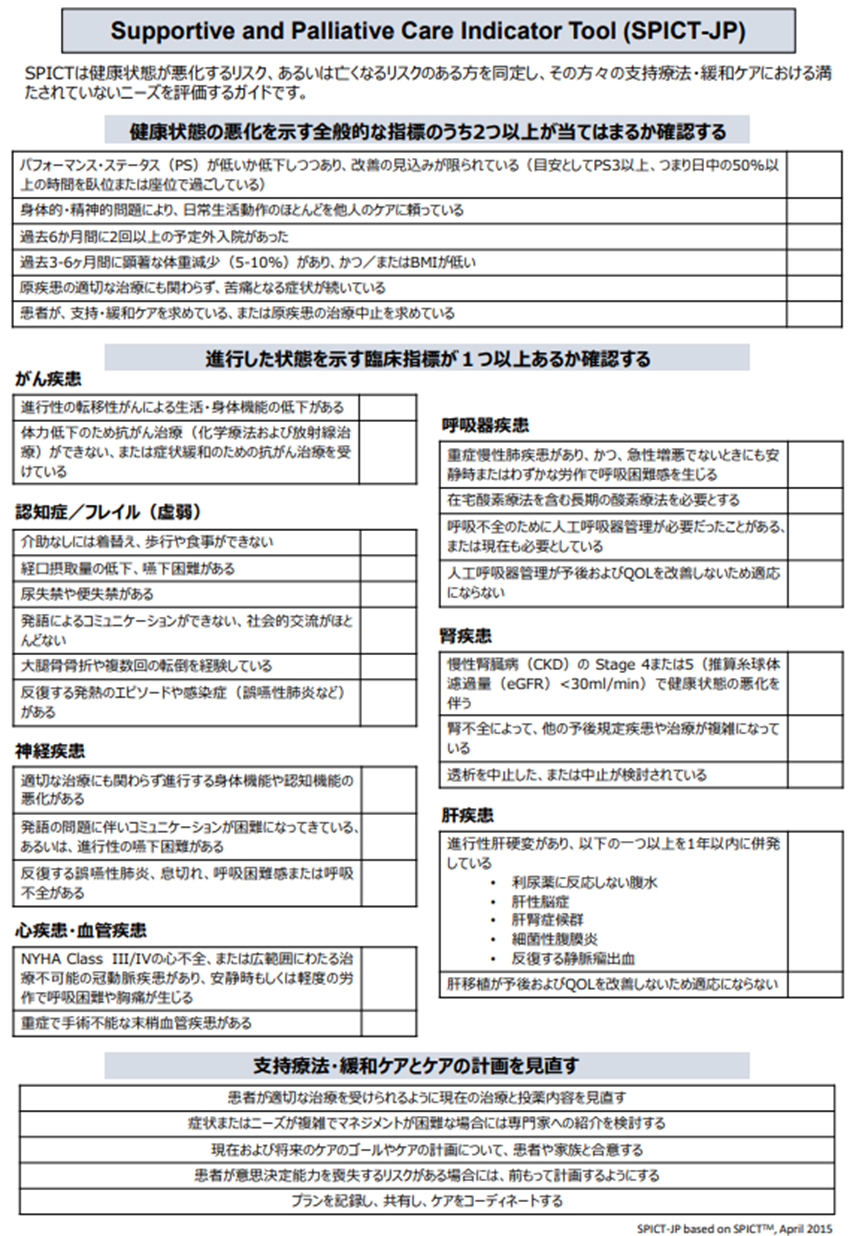


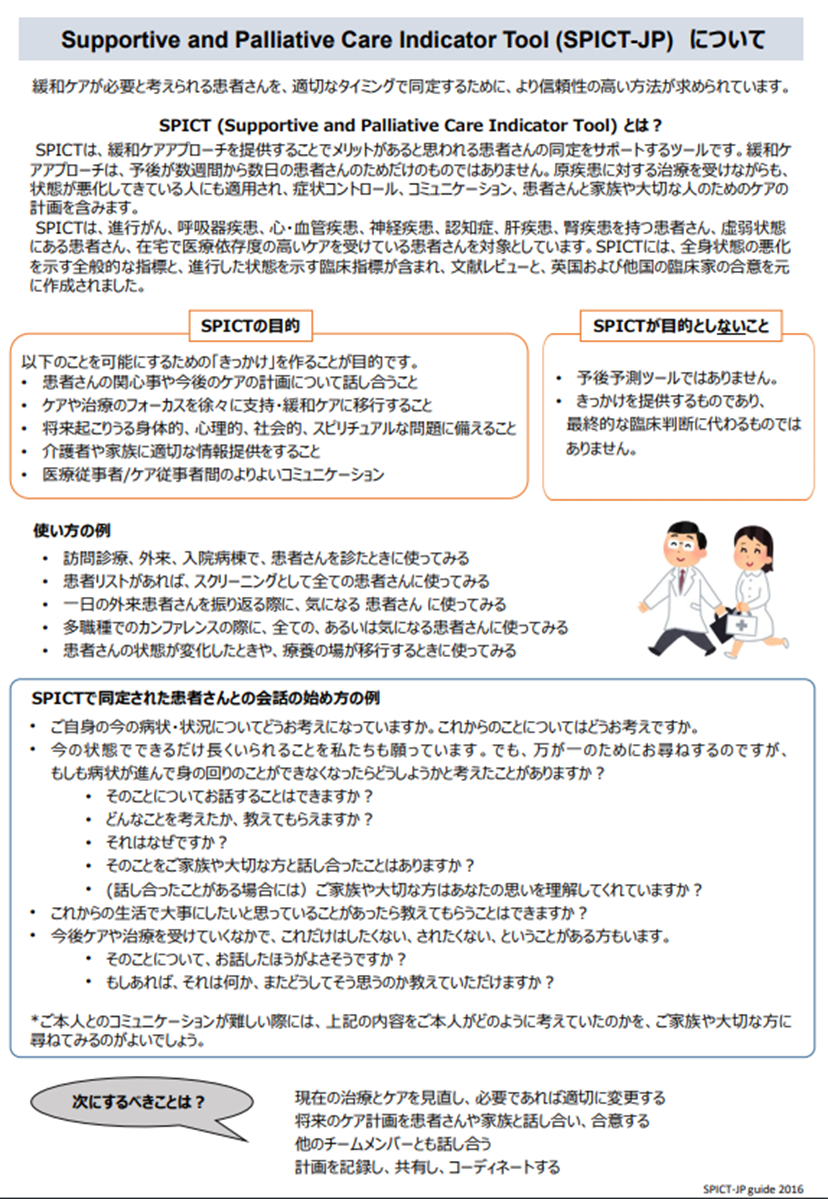

Supplement: Supplemental data [file Supp_DataS5.docx]
