# Supplementary material for: Translation and Cross-Cultural Adaptation of the Supportive and Palliative Care Indicators Tool into Japanese: A Preliminary Report
Source: Palliat Med Rep. 2022 Aug 18;3(1):1–5. doi: 10.1089/pmr.2021.0083 (PMC9438437; doi:10.1089/pmr.2021.0083)
Supplement: Supplemental data [file Supp_DataS4.docx]

**Supplement 4. Feedback from the expert committee review on the SPICT-JP**

**Who is considered as SPICT-JP positive/negative?**

Firstly, there were some comments regarding instructions in the SPICT-JP. It was unclear for some members when patients were considered as SPICT ‘positive’. For example:

*It’s not easy to understand how to use this tool. If the patient meets two or more general indicators or one or more clinical indicators, then should we review the patient? It would be better to have an explanation on how to use it at the beginning.* (Expert committee member No.2)

**Unclear criteria within the tool**

Expert committee members identified some unclear criteria within the tool, such as ‘low BMI’ or ‘severe’ diseases. Developers had intentionally left these criteria vague so that users could act intuitively when making their decisions in accordance with patients’ situations, and to allow the SPICT tool to be applicable to community settings where specific test results may not be available. However, some members thought that a ‘tool’ should have a clear cut-off and not allow users to act intuitively.

**Who should use the SPICT-JP?**

Some members raised the issue of the position of the SPICT-JP within the Japanese healthcare system. One member believed incorrectly that the original SPICT was only for GPs in the UK. He claimed that the lack of family physicians and the less-established primary care in Japan might impede the implementation of the SPICT-JP. For example:

*In the UK, GPs, who are looking at patients holistically, would undertake the role of evaluating their patients regarding indications for supportive and palliative care needs and provide them accordingly. However, in Japan, many such patients are seen by hospital specialists in the first instance, and these specialists are only looking at organs within their speciality. It is probably difficult for them to use this tool in hospital. It is difficult for me to imagine in what situations this tool would be used in Japan.* (Expert committee member No.6)

One member thought that patients who needed to be evaluated for palliative care needs were seen by hospital doctors rather than family physicians in Japan. Similarly, another member argued that doctors or hospital specialists – other than family physicians – might be best placed to use the SPICT-JP on the basis that hospital doctors rather than family physicians would see people needing palliative care more often. Conversely, another comment from a panel member indicated that family physicians were better placed to use the tool because they were generally better at introducing discussions about end-of-life care.

*It would be better to promote and cascade this (SPICT-JP) to organ-specific specialists and co-medicals [allied health professionals] working in their specialities. It would make appropriate supportive and palliative care accessible to many more people.* (Expert committee member No.4)

*Family physicians, in general, tend to have advanced care planning (ACP) in their mind when seeing patients, and most frail elderly patients would meet these criteria, I would question the value of using this tool (It would be easy to suggest we should consider ACP with all frail elderly patients without using this tool). On the other hand, it might be useful as a tool for junior doctors or family medicine trainees to remind them of ACP, or to flag up to them the situations when ACP should be considered. It might be more valuable for organ-specific specialists to use the tool as they tend to concentrate on organ-specific treatments [and do not take a holistic approach] compared to family physicians.* (Expert committee member No.8)

**Lack of awareness of palliative care and end-of-life issues**

There were some comments regarding awareness of palliative care among the general public and healthcare professionals. One member stated:

*In addition, the concept of palliative care is not common in Japan as yet. The problem is that not only opioids but other resources for palliative care are limited for non-cancer patients*. (Expert committee member No.6)

He also suggested that the ‘not-telling the truth’ culture would interfere with providing palliative care as well as identifying patients needing palliative care in relation to one specific item (request for palliative care) in the SPICT-JP.

*In order to respond to the item ‘patient asks for supportive and palliative care, or treatment withdrawal’, patients should have been informed of their medical conditions and prognosis. But in Japan, there are many cases in which only families are informed. I mean, we need to evaluate if the patients are well informed to make it possible for them to make their own decisions.* (Expert committee member No.6)

Interestingly, the same doctor wrote the following comment, which in a sense showed that he did not connect end-of-life discussions with palliative care.

*Combining these general indicators, bedbound people – due to cerebral infarction, for example – would be indicated as needing supportive and palliative care as well, but many of these people would be stable for more than ten years. I cannot imagine what palliative care is like for these people, including dementia.* (Expert committee member No.6)

Concerning general public awareness, one member suggested that only a few patients were ready to accept palliative care when they needed it. He explained that to relieve their anxiety of being abandoned, a careful review of care and treatment was of significant importance.

*It would be possible to identify people with ‘indications’ for supportive and palliative care by using SPICT-JP. However, the issue is to what extent patients feel their ‘needs’ for supportive and palliative care. This is the case, particularly for non-cancer patients. Even if these patients themselves don’t wish for so-called resuscitation or artificial ventilation, their families request for artificial nutrition or specialist treatment for acute exacerbations which sometimes makes the gradual transition to supportive and palliative care difficult.* (Expert committee member No.4)

*It is very important to review carefully and sensitively the current treatments and care plans rather than promoting the advance are planning to prevent them from feeling abandoned* [by medical professionals] *or any misunderstandings by patients regarding the lack of necessary treatments just because they are too old. And, it would make it easier for patients and families to agree on appropriate treatment and care.* (Expert committee member No.4)

Similarly, another member indicated that not many Japanese doctors were good at reviewing, discussing and communicating patients’ care plans. He suggested that there would be many patients who suffered from being treated without having their wishes heard or respected.

*Many Japanese doctors are not good at carrying out the five reviews* [i.e. the five bullet points in the care planning box in the SPICT-JP]*, reviewing the treatment and drugs, referring patients to palliative care or the introduction of palliative care, and advance care planning (It’s not only the doctors’ problem, but it has a lot to do with society). Is it only me to feel these reviews are still valuable? It’s valuable if we could recognise that many more people would benefit from being reviewed. This would help those patients who are only receiving treatment and thus suffering*. (Expert committee member No.5)

**‘Effectiveness’ of the SPICT-JP**

Another issue raised by the expert committee members was the ‘effectiveness’ of the tool. Several members commented that it would be useful if the information is made available regarding the ‘effectiveness’ of the tool or potential benefits of using the tool in their practice.

*It would be much better if any data about the effectiveness of the SPICT were made available. It would motivate us to use it*. (Expert committee member No.8)

These opinions were related to the absence of an explicit purpose for the SPICT-JP and of the contexts for SPICT-JP use within the Japanese primary care setting.

*It’s not clear what one would like to do with this tool and what would be the benefits of using this tool. Is this a checklist, not a prognostic tool? Has anyone achieved a better quality of care by using the SPICT? It would be easy to understand if it provides a score, and that score indicates some specific action. It’s not clear what one would like to achieve and how this would be useful. Providing information on the effectiveness and the possible usage of the tool would make it more attractive.* (Expert committee member No.7)

*In our hospital, we advocate ‘palliative care for all’. I mean we separate specialist and basic palliative care to some extent. But is the SPICT for specialist palliative care (for the severely ill)?* (Expert committee member No.5)

These problems might be resolved by providing more information on the potential usefulness of the SPICT-JP in the Japanese primary care setting. On the other hand, there is little evidence for its ‘effectiveness’. Furthermore, the best way to use the SPICT-JP in Japanese healthcare settings remains unknown, which our further research aims to address. Therefore, it was impossible to provide detailed information about the effectiveness or ultimate goals of using the SPICT-JP in Japanese contexts during the translation and adaptation process.
